# ASMPKS: an analysis system for modular polyketide synthases

**DOI:** 10.1186/1471-2105-8-327

**Published:** 2007-09-03

**Authors:** Hongseok Tae, Eun-Bae Kong, Kiejung Park

**Affiliations:** 1 Information Technology Institute, SmallSoft Co., Ltd., Jang-Dong 59-5, Yusung-Gu, Daejeon 305-343, South Korea; 2 Deptartment of Computer Engineering, Chungnam National University, 220 Gung-dong, Daejeon 305-764, South Korea

## Abstract

**Background:**

Polyketides are secondary metabolites of microorganisms with diverse biological activities, including pharmacological functions such as antibiotic, antitumor and agrochemical properties. Polyketides are synthesized by serialized reactions of a set of enzymes called polyketide synthase(PKS)s, which coordinate the elongation of carbon skeletons by the stepwise condensation of short carbon precursors. Due to their importance as drugs, the volume of data on polyketides is rapidly increasing and creating a need for computational analysis methods for efficient polyketide research. Moreover, the increasing use of genetic engineering to research new kinds of polyketides requires genome wide analysis.

**Results:**

We describe a system named ASMPKS (Analysis System for Modular Polyketide Synthesis) for computational analysis of PKSs against genome sequences. It also provides overall management of information on modular PKS, including polyketide database construction, new PKS assembly, and chain visualization. ASMPKS operates on a web interface to construct the database and to analyze PKSs, allowing polyketide researchers to add their data to this database and to use it easily. In addition, the ASMPKS can predict functional modules for a protein sequence submitted by users, estimate the chemical composition of a polyketide synthesized from the modules, and display the carbon chain structure on the web interface.

**Conclusion:**

ASMPKS has powerful computation features to aid modular PKS research. As various factors, such as starter units and post-processing, are related to polyketide biosynthesis, ASMPKS will be improved through further development for study of the factors.

## Background

As many infectious microorganisms have been acquiring tolerance to antibiotics, the need for novel antibiotics is increasing. Modification of known antibiotics is a more efficient approach than finding microorganisms with new kinds of antibiotic activities, and various methods are being used to develop novel antibiotics, including gene manipulation for biosynthesis of antibiotics such as polyketides.

Polyketides are secondary metabolites of many kinds of microorganisms [1,2] with diverse biological functions, including pharmacological activities such as antibiotic, antitumor and agrochemical properties. While the polyketide antibiotics are important clinical drugs such as avermectin [3,4] and erythromycin [5], new kinds of polyketides are still being discovered. Polyketides are synthesized by serialized reactions of a set of enzymes called polyketide synthase(PKS)s [6], which coordinate the elongation of carbon skeletons by the stepwise condensation of short carbon precursors [7]. PKSs are classified into two types, depending on the organization of their active sites, namely modular [8,9] and iterative [10,11] PKSs. Modular PKSs are multifunctional enzymes composed of so-called modules [12,13], which noniteratively process serialized steps of polyketide chain elongation. Iterative PKSs are multiple enzyme complexes that iteratively perform a set of activities used in various condensation and chain elongation steps. PKS genes that synthesize a polyketide are usually clustered on a genome, and so the chemical structure of the polyketide can be modified through gene manipulation [14,15]. For example, the chemical structure of a polyketide can be modified by changing the genes related to starting units [16,17], extender units [18], the reduction of carbonyl carbons [19], the length of the carbon chain [20], or post-processing. In addition, genetic engineering, whether by the fusion of different polyketides [21] or their biosynthesis in heterologous hosts [22], has been shown recently to improve polyketide activities.

Although polyketides have been shown to be important antibiotics, few computational analysis systems have been developed. A lack of adequate polyketide databases has caused researchers to study many chemical databases to collect polyketide data. Computational analysis for polyketide data can reduce the time and the cost of the research. PKSDB [23], developed by the National Institute of Immunology (NII) of India, contains data on 20 modular polyketides. While it has some visualization and analysis components, more features and functions are needed, including an efficient database system to manage and analyze polyketide data.

Catalytic domains, which organize PKS modules with their linkers [24], are defined according to their function. The core domains related to carbon chain elongation include an acyltransferase domain (AT), which selects and transfers extender units; an acyl carrier protein (ACP), which tethers the growing polyketide chain to the PKS for condensations; and a ketoacyl synthase (KS) domain, for decarboxylative condensations. Additional domains participate in the modification of the carbonyl group; these include ketoreductase (KR), dehydratase (DH) and enoyl reductase (ER) domains. A thioesterase (TE) domain catalyzes the release of the polyketide product from the last PKS participating in chain elongation. We have developed ASMPKS (an Analysis System for Modular Polyketide Synthesis) to efficiently support computational analysis of the modular PKS in genome sequences. ASMPKS operates as a web application and provides various features including visualization of polyketide structures, new PKS assembly simulation and management of the modular polyketide database. Homology search, multi-alignment of domains and polyketide prediction are also available. BLAST [25] and ClustalW [26] were used for the homology search and multi-alignment, respectively. An important feature of ASMPKS is its genome analysis component, which finds known PKS clusters and predicts putative PKS clusters in given microbial genome sequences. This component analyzes several genomes and stores the result in the database, which is then displayed by the genome browser. PKS analysis against genome sequences can make it very easy to find known PKSs and to select novel candidate PKS genes.

## Implementation

### PKS search against microbial genome sequences

ASMPKS searches microbial genome sequences for modular PKSs. It detects PKS gene clusters producing known polyketides, which are included in the database, by measuring the homology between protein sequences of an annotated genome and all PKS sequences, and predicts unknown gene clusters to produce putative polyketide candidates by identifying domains. It can accept genome sequences or GenBank format data, including gene information. If genome sequences are submitted, genes are predicted by Glimmer [27], and their sequences are converted to proteins.

**Figure 1:**
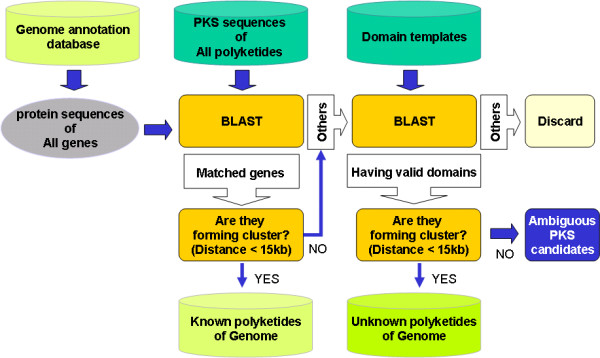
**The protocol of automated polyketide annotation for a genome**. ASMPKS searches microbial genome sequences for modular PKSs. It detects PKS clusters producing known polyketides by measuring the homology between protein sequences of an annotated genome and all PKS sequences, and predicts unknown gene clusters to produce putative polyketide candidates by identifying domains.

The automated genome wide PKS analysis consists of two processes, which find known and unknown PKS gene clusters, respectively (Figure 1). The first process, to detect known PKS gene clusters, begins by measuring the homology between protein sequences of an annotated genome and all PKS sequences in the database with the BLAST program. If the e-value of homology between two sequences is 0.0 and the alignment length is more than 90 percent of the matched PKS, the query protein is considered as making the same product as the PKS. If corresponding proteins to all PKSs for a polyketide exist and they form a gene cluster with some distance measures, the cluster is marked as producing the polyketide.

In the second process, the remaining proteins are aligned with template sequences of KS, AT, DH, ER and KR domains, each of which has the highest similarity with other sequences belonging to the same domain type. From the result of BLAST, the fragments with e-values less than 0.000001 are taken as domains, and the proteins with valid domain compositions, at least 'KS-AT-ACP', are selected. If the distance between neighbouring proteins is less than 15 kilobases, they belong to the same cluster and the cluster is marked as producing a new kind of polyketide. If a protein has domains and does not belong to a cluster, it is marked as an ambiguous PKS candidate.

### Domain identification from protein sequences

ASMPKS predicts domain information from protein sequences. Domain identification is based on the homology search method with template sequences of domains. To detect domains, BLAST is used. Template sequences that represent each domain type are formatted into the BLAST database file. To select each template sequence representing each domain type, homology scores between every pair of sequences of that type are measured, and the sequence with the highest score, which is the sum of its top 10 homology scores with other sequences, is selected. KS and AT from module 8 of amphotericin [28], DH from module 5 of nystatin [29], KR from module 15 of nystatin, ER from module 15 of amphotericin, ACP from module 3 of nystatin and TE from module 6 of erythromycin [5], were selected.

The accuracy of ASMPKS in identifying domains from protein sequences was tested with three groups. The group 1 contains the PKS information for the polyketides of PKSDB[23] which includes 906 domains of the 20 polyketides. The group 2 is composed of 792 domains included in the modular PKSs of 17 type I polyketides. And the group 3 is composed of 129 domains of 5 PKS-NRPS (nonribosomal peptide synthetases) hybrid types. The group 1 was used to construct the initial ASMPKS database and prepare template sequences of domain types. And the test accuracy to search each group against the initial ASMPKS database was evaluated (Table 1). ASMPKS showed high accuracy for the three groups. It has a specificity of 99.7% and a sensitivity of 98.1% for the group 2 which includes domains of modular PKSs and for the group 3 it shows somewhat low sensitivity (85.2%) especially in the DH domains, for which ASMPKS identified 5 of 13. Currently the information of the group 2 is also added to the database of ASMPKS.

**Table 1:** Accuracy of ASMPKS in the domain identification

	No of total	No of predicted	No of correct	SP(%)	SN(%)
**Group 1**	906	900	899	99.8	99.2
**Group 2**	792	779	777	99.7	98.1
**Group 3**	129	111	110	99.1	85.2

**Specificity **(SP) = Correct predictions/Total predictions,

**Sensitivity **(SN) = Correct predictions/Total domains

The group 1 is composed of 906 domains of 20 polyketides, amphotericin, ascomycin, avermectin, epothilone, erythromycin, megalomicin, myxalamid, myxothiazol, niddamycin, nystatin, oleandomycin, pikromycin, pimaricin, pyoluteorin, rapamycin, rifamycin, soraphen, spinosad, stigmatellin, and tylactone, that are included in PKSDB.

The group 2 is composed of 792 domains in modular PKSs of 17 type I polyketides which are aureothin, borrelidin, bryostatin, concanamycin a, coronatine, fk506, fr-008, geldanamycin, herbimyycin a, lankamycin, methymycin, monensin, mycolactone, nanchangmycin, phoslactomycin, rubradirin and vicenistatin.

The group 3 is composed of 129 domains of 5 PKS-NRPS hybrid types which are bacillaene, cryptophycin, lankacidin, leinamycin and pederin.

## Results and discussion

### Database of ASMPKS

ASMPKS has been developed for the overall management of modular PKS data. As it operates on the web interface to construct the database and to analyze polyketides, the extension of database is very accessible for multiple users. Researcher can add and delete their data in the database easily. The database system of ASMPKS is divided into two parts. The first part, which has been constructed with published data, contains information regarding PKS genes, modules, domains and assembly. It is used to search and to align domains of protein sequences. The second part, which contains genome data of microorganisms and polyketide information related to the genomes, allows researchers to study synthesis of polyketides in a specified microorganism.

The ASMPKS database includes the PKSDB data, and more entries are being added by an updating interface. ASMPKS searches domain and module data against genome sequences, reports known PKS clusters producing known polyketides and predicts unknown gene clusters of putative polyketide candidates. The analysis results are stored in the database to be used in the next PKS analysis.

### Visualization of detailed polyketide information

**Figure 2:**
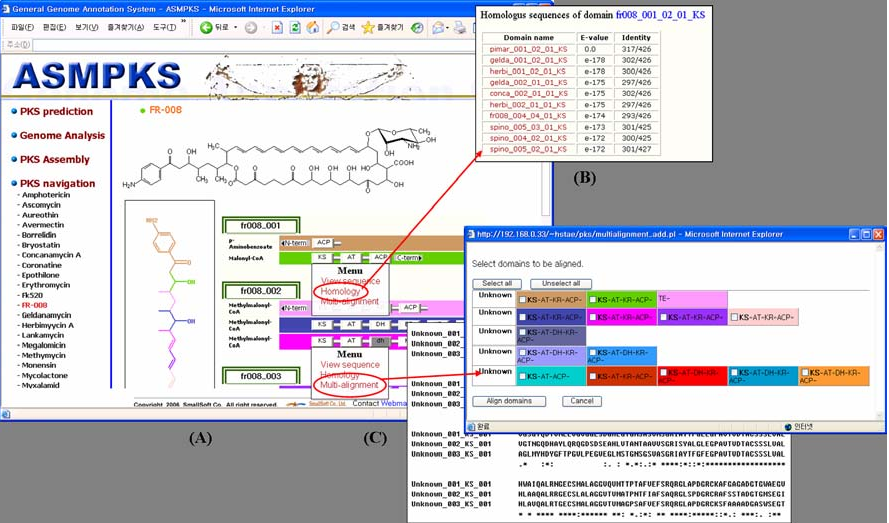
**Information viewers for a polyketide**. (A) ASMPKS shows the structure and PKS composition of a selected polyketide, and provides (B) domain homology search and (C) multiple domain alignment.

PKSs consist of sets of modules, and each module contains two or more functional domains and processes one chain condensation reaction to synthesize a polyketide. The PKS composition and the chemical structure of a polyketide in the database are displayed by the PKS navigation component (Figure 2A), which shows the arrangements of the PKSs with their domain composition and draws the intermediate chain for a selected polyketide.

The domain button has a hyperlink to homology search and multiple sequence alignment components for the analysis of domain similarity relationships. BLAST is used for homology analysis between the same type domains. When a domain is selected, ASMPKS uses BLAST to search all domains of the same type in the database and displays a list of all homologous domains (Figure 2B). ClustalW is used for multiple sequence alignments. When a domain type is selected, all domain sequences of the type in the database can be added to the list to be aligned (Figure 2C). These two similarity analysis features are also applied to the domains of unidentified PKSs in domain identification and genome analysis components. Researchers can compare their polyketide data with the public data in the ASMPKS and predict the structure or activity of their novel polyketides.

### Simulation of the assembly for predicted PKSs and construction of an expected chain

If the module sequence of PKSs for a predicted polyketide is completed, the carbon body of the polyketide can be predicted. The chemical structure of a polyketide makes its chemical activity easily understood. The ASMPKS provides a PKS assembly component, which assembles a set of modules and shows the construction of an expected carbon body for a predicted polyketide. The biosynthesis of a polyketide begins by selecting a starter unit and continues by adding many extender units onto the carbon chain until a TE domain appears. As there are various kinds of starter and extender units, diverse polyketides can be constructed by their combination. In this version, the starter units include 2-Methylbutyryl-CoA, 3,4-DHCHC-CoA, 3,5-AHBA-CoA, 3-Amino-2-Methylpropionate, 3-Methylbutyryl-CoA, Acetoacetyl-CoA, Acetyl-CoA, Benzoyl-CoA, Butyryl-CoA, Cyclohexanecarboxylic acid, Glycine, Glycolate, p-Aminobenzoate, p-Coumaroyl-CoA, p-Nitrobenzoate, Propionyl-CoA and trans-1,2-CPDA, and the extenders include malonyl-CoA, methylmalonyl-CoA, Ethylmalonyl-CoA and Hydroxymalonyl-CoA, both of which are stored in the database. The feature to adding new starter and extender units to the database via the user interface will be available in ASMPKS.

**Figure 3:**
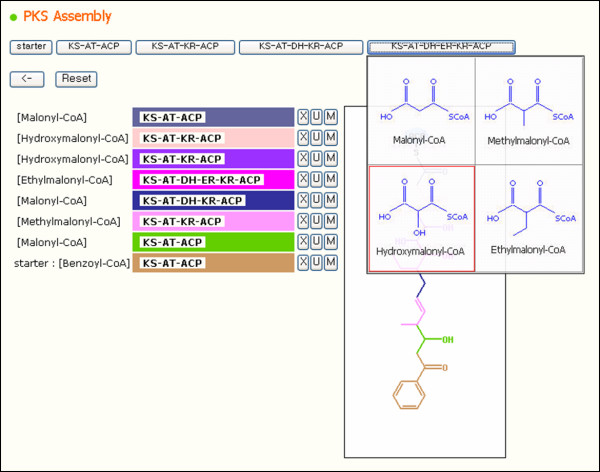
**Features for PKS assembly**. Users can assemble a novel PKS by selecting modules and units, and the expected polyketide chain produced by the assembled PKS is shown.

To simulate a new combination of modules, the starter unit should be selected first. When the 'starter' button is clicked, starter units stored in the database are listed (Figure 3). Users can select a starter unit and append extender units, such as malonyl-CoA and methylmalonyl-CoA, by clicking the buttons on which 'KS-AT-ACP', 'KS-AT-KR-ACP', 'KS-AT-DH-KR-ACP' or 'KS-AT-DH-ER-KR-ACP' is written. The labels written on the buttons represent the main modules of PKSs. Each module processes the extender unit with its catalytic domains and adds it to the carbon chain. The structure viewer of the component shows an expected carbon body anchored to an ACP domain. Each colored part on the carbon chain is the product of its module listed on the left table in the figure. This component can be used to predict the structure of a new polyketide.

### PKS visualization on a genome browser

**Figure 4:**
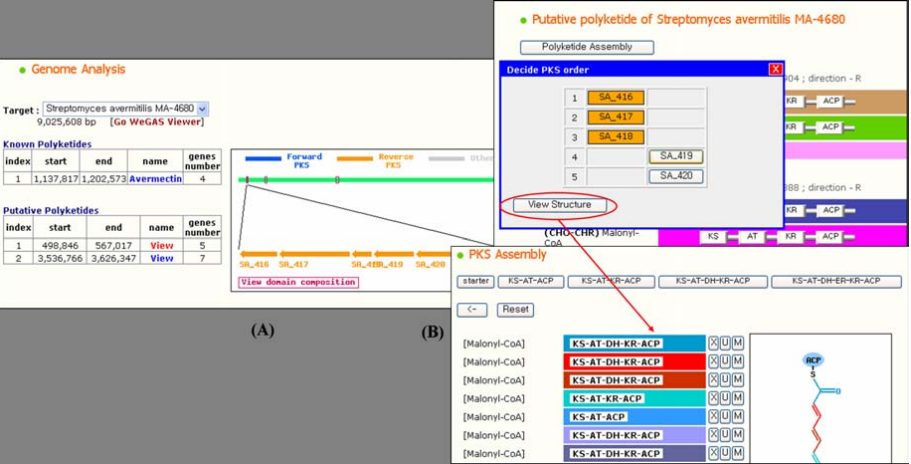
**PKS analysis result for genome sequences**. (A) The result of the PKS analysis process against microbial genome sequences can be displayed on the genome browser. The listed polyketides of the genome on the left side are divided into two groups, known and putative. (B) Users can set the order of PKSs and see the intermediate chain of a putative polyketide.

The result of the PKS analysis process against microbial genome sequences can be displayed in the genome browser of ASMPKS (Figure 4). It shows the position and composition of gene clusters of each polyketide on a genome. The displayed polyketides of the genome are divided into two groups, known and putative. The known polyketides are entities of the database of ASMPKS and the putative polyketides do not belong to the database but are predicted by the PKS search feature. A core chain of the putative polyketide can be shown by the PKS assembly module. ASMPKS assigns substances, such as malonyl-CoA or methylmalonyl-CoA, to AT domains in the PKSs of a putative polyketide according to their motif related to the substances, and generates an expected polyketide chain by manual ordering of PKSs.

The detailed genome and gene information can be shown by our genome annotation system called WeGAS[30]. The integration of two systems aids researchers to get useful data of PKSs without searching public databases such as NCBI.

### Updating polyketide entry data of the database

**Figure 5:**
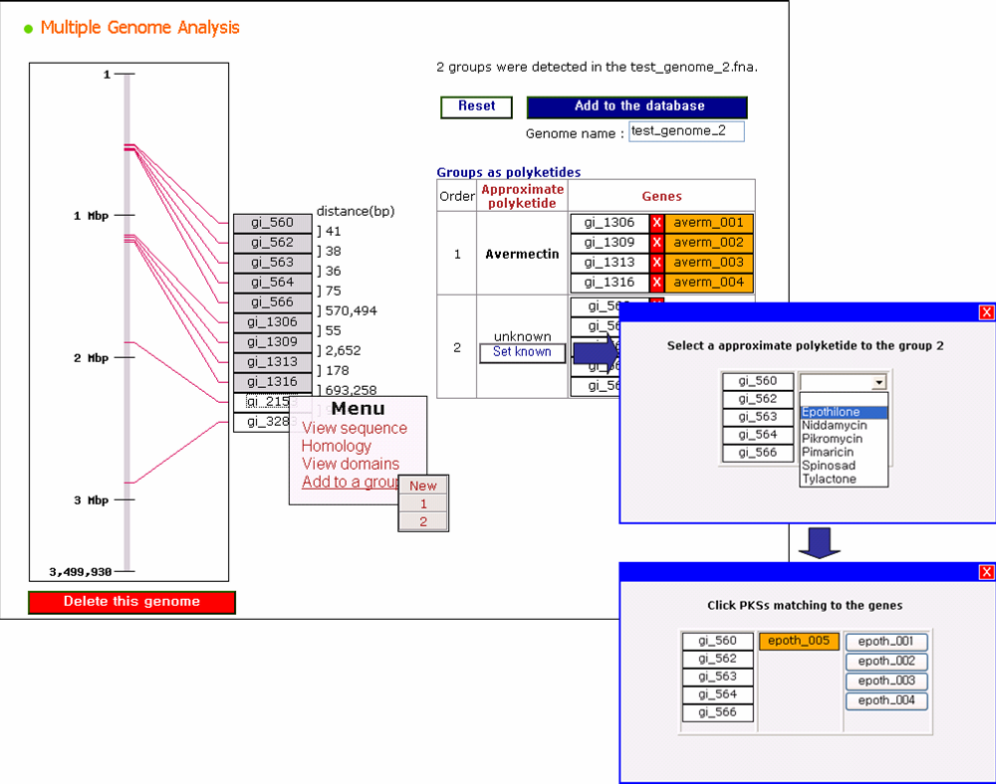
**The management component for the genome data update**. Using the automated polyketide annotation process, managers can edit the result by reforming gene clusters and/or annotating the clusters as producing known polyketides, and/or uploading it to the database.

ASMPKS provides methods to update polyketides and genome annotation data through web interfaces. To update polyketide data, it supports the update interface for all information of a polyketide, including a chemical structure image and PKS sequences. For the submitted PKS sequences, it suggests the optimal composition of domains and units for the PKSs. Users can edit the domain composition and insert it to the database. With the inserted data, ASMPKS shows the intermediate carbon chain produced by the PKSs. To update genome annotation data, the automated polyketide annotation process should be performed, after which it can be edited and updated by system managers. The PKSs found in the process are divided into three groups: 1) PKSs producing known polyketides present in the ASMPKS database, 2) PKSs not included in the database but making clusters on the genome sequence, and 3) PKSs not included in any cluster. On the editing page, all predicted PKSs are displayed according to their position and marked with their groups. They can be added to or removed from a PKS cluster, and putative polyketides can be annotated as known polyketides (Figure 5). The edited result is stored in the database and displayed in the genome browser.

## Conclusion

ASMPKS has been developed for computational analysis of the modular PKS for genome sequences. It also provides overall management of information on modular PKS, including PKS database construction, new PKS assembly, and visualization of polyketide structures. It is a useful system to analyze known polyketides and to predict new polyketides. The PKS assembly and genome analysis components are especially powerful computation tools for polyketide research.

As various factors are related to polyketide biosynthesis, ASMPKS can be improved through further study. Biological activities of many polyketides are critically related to post-synthetic processing steps, such as O-methylation, alkylation, cyclization and glycosylation, which give polyketides diverse attributes. ASMPKS will include the information and analysis features for post-synthetic processing. In addition, while the current version constructs only the carbon body under the PKS assembly component, the procedure displaying a completed chemical structure of an expected polyketide from the new module composition will be added later. With the addition of new features, it will aid in more efficient research on polyketides. ASMPKS is a system to analyze modular PKSs, and it does not provide the analysis features for other PKS types. Because many polyketides are produced by PKS-NRPS hybrid enzymes or iterative PKSs, the analysis components for these types are necessary. ASMPKS will be improved as an overall management system of polyketides including other PKS types.

## Availability and requirements

Project name: modular PKS analysis system

Project home page: 

Operating system(s): Linux

Programming language: Perl

Other requirements: Apache web server, Mysql 4.1 or higher, GD-2.30 or higher

Any restrictions to use by non-academics: yes, contact the author HT for details.

## Abbreviations

PKS: polyketide synthase; KS: ketoacyl synthase; AT: acyltransferase;

ACP: acyl carrier protein; KR: ketoreductase; DH: dehydratase; ER: enoyl reductase;

TE: thioesterase; NRPS: nonribosomal peptide synthetases

## Authors' contributions

HT developed the software and EBK revised the manuscript. KP guided the development of this project. All authors have read and approved the final manuscript.
